# Beagle dog 90-day oral toxicity study of a novel coccidiostat – ethanamizuril

**DOI:** 10.1186/s12917-020-02655-2

**Published:** 2020-11-17

**Authors:** Keyu Zhang, Haihong Zheng, Shuya Wei, Xiaoyang Wang, Chenzhong Fei, Chunmei Wang, Yingchun Liu, Lifang Zhang, Feiqun Xue, Shusheng Tang

**Affiliations:** 1 Key Laboratory of Veterinary Chemical Drugs and Pharmaceutics, Ministry of Agriculture and Rural Affairs, Shanghai Veterinary Research Institute, Chinese Academy of Agricultural Sciences, Shanghai, 200241 People’s Republic of China; 2 School of Biological Engineering, Wuhan Polytechnic, Wuhan, 430074 People’s Republic of China; 3 College of Veterinary Medicine, China Agricultural University, Beijing, 100193 People’s Republic of China

**Keywords:** Ethanamizuril, Oral subchronic toxicity, Beagle dogs, Triazine coccidiostats, Nephrotoxicity

## Abstract

**Background:**

Triazine coccidiostats are widely used in chickens and turkeys for coccidiosis control. Ethanamizuril is a novel triazine compound that exhibits anticoccidial activity in poultry. This study was designed to evaluate the subchronic toxicity of ethanamizuril in beagle dogs at doses of 12, 60 or 300 mg/kg/day in diet for 90 days.

**Results:**

Ethanamizuril was well tolerated at low and middle dosages in beagle dogs, and no drug-related toxical effects were observaed in terms of survival, clinical observations, organs weight and damage in these dose groups. However, in high dose administration group, food consumption and histologic changes in kidneys were noticed in both sexes of beagle dog, although the renal lesions were finally resolved at the end of 4 weeks exposure of ethanamizuril.

**Conclusions:**

No-observed-adverse-effect level (NOAEL) was considered for ethanamizuril at dose of 60 mg/kg/day in Beagle dog. This result added toxicity effects of ethanamizuril to the safety database, which might guide safely using of ethanamizuril as a novel coccidiostat.

**Supplementary Information:**

The online version contains supplementary material available at 10.1186/s12917-020-02655-2.

## Background

Coccidiosis is a detrimental disease of the intestinal tract of animals caused by several species of Eimeria protozoa. In the commercial broiler industry, Eimeria protozoan is parasitizing on the intestinal epithelium of chicken, which led to considerable impairment of growth and feed utilization, and resulted in significant impact on mortality and morbidity. Coccidiosis is able to inflicte devastating economic losses to poultry operations. It is estimated that the substantial economic burden caused by avian coccidiosis was more than $ 3 billion annually to the industry worldwide [1, 2]. Lack of safe and effective coccidiosis vaccines, commercial synthetic anticoccidial drug has been used as the main strategy for control of coccidiosis since the late 1940s [3]. However, more and more clinical studies showed the widespread resistances to commercial anticoccidial drugs have emerged in coccidian parasites due to misuse of anticoccidial drugs [2, 4]. Consequently, the resulting of letdown treatment suggests a constant demand for innovative products with safe and efficient. Triazines are benzene-aceto-nitrile compounds including diclazuril, toltrazuril, ponazuril and clazuril, which have been used globally in the interception and therapy of protozoal diseases, most commonly in coccidiosis of veterinary interest since 1980s [5]. However, the widely use of triazine coccidiostats have generated resistance to the genus Eimeria in recent years [5, 6]. Fortunately, no cross-resistance was observed in triazine coccidiostats [5].

**Fig. 1:**
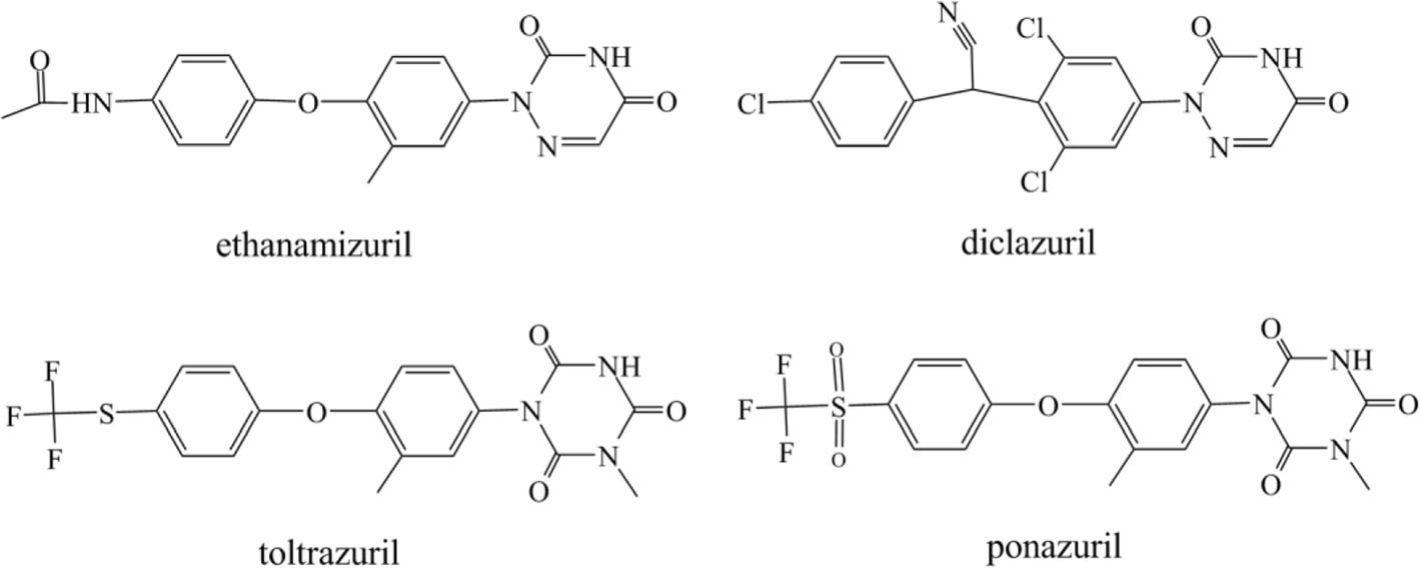
Chemical structure of triazine coccidiostats, including ethanamizuril, diclazuril, toltrazuril and ponazuril

Ethanamizuril (Fig. 1), a novel triazine coccidiostat, namely (N-(4-(4-(3,5-dioxo-4,5-dihydro-1,2,4-triazin-2-(3H)-yl)-2-methylphenoxy) phenyl)acetamide, has been independently established by the Shanghai Veterinary Research Institute of the Chinese Academy of Agricultural Sciences in recent years and exert wide application prospective in future [5, 7]. In China and Japan Patents, the chemical synthesis of ethanamizuril and the chemical structure of this compound have been published [8]. Ethanamizuril has displayed excellent efficacy against Eimeria protozoa such as *Eimeria tenella, Eimeria. necatrix, Eimeria. acervulina,* and *Eimeria*. *maxima* in broiler chickens. Typical dosage was reported as 10 mg/kg in the feed or 10 mg/l in the drinking water [7, 8]. At the recommended dose, ethanamizuril could significantly improve feed conversion ratios and live weight, reduce oocyst excretion, and also decrease mortality and lesions in broilers. ACIs (anticoccidia indexes) of ethanamizuril could be reached to as high of 185 ~ 190. Furthermore, the preclinical pharmacodynamic studies demonstrated ethanamizuril did not cause cross-resistance with diclazuril or toltrazuril resistant *Eimeria. tenella* in broilers.

Because of the diverse toxicities, the use of some coccidiostats, such as arprinocid, roxarsone and arsanilic acid has been forbidden in poultry industry [9, 10]. Up to now, a series of studies have been conducted to evaluate the safety of diclazuril and toltrazuril for use as coccidiostats. Increase of liver weight and swelling of the centrilobular hepatocytes were seen in diclazuril treated mice and rats [11–13]. The slight effects on haematological parameters, disturbances of the liver function and decreased on the weight gain and the daily feed intake were observed in toltrazuril treated rats [11, 12, 14]. In addition, in two teratogenicity studies, teratogenicity and embryotoxicity were observed at the toltrazuril highest dose in rats [14, 15]. With high anticoccidial effectiveness, ethanamizuril would be used widely in poultry industry in future. A series of toxicity evaluation of ethanamizuril has been carried out in rats and mice, and the NOAEL for the dietary administration of ethanamizuril in the 90-day oral toxicity study for rats was greater than 20 mg/kg. According to VICH’s guidelines, the dog is the default non-rodent species required for repeat-dose toxicity testing [16]. However, to the best of our knowledgee, the toxicity evaluation in beagle dogs has not been reported. In order to gain our understanding of the toxicity for ethanamizuril, we conducted a 90-day toxicity study of ethanamizuril in beagle dogs as following guidelines of veterinary safety evaluation.

## Results

### Survival and clinical observations

No unscheduled mortality occurred during the study. During the experimental period, there were no ethanamizuril related clinical signs of toxicity observed, and no abnormal behaviour or altered activities were noted. All observed phenomena in the study period were normal for laboratory dogs of the breed and age. Ophthalmological examinations did not find ethanamizuril related changes.

### Food consumption and body weights

**Fig. 2:**
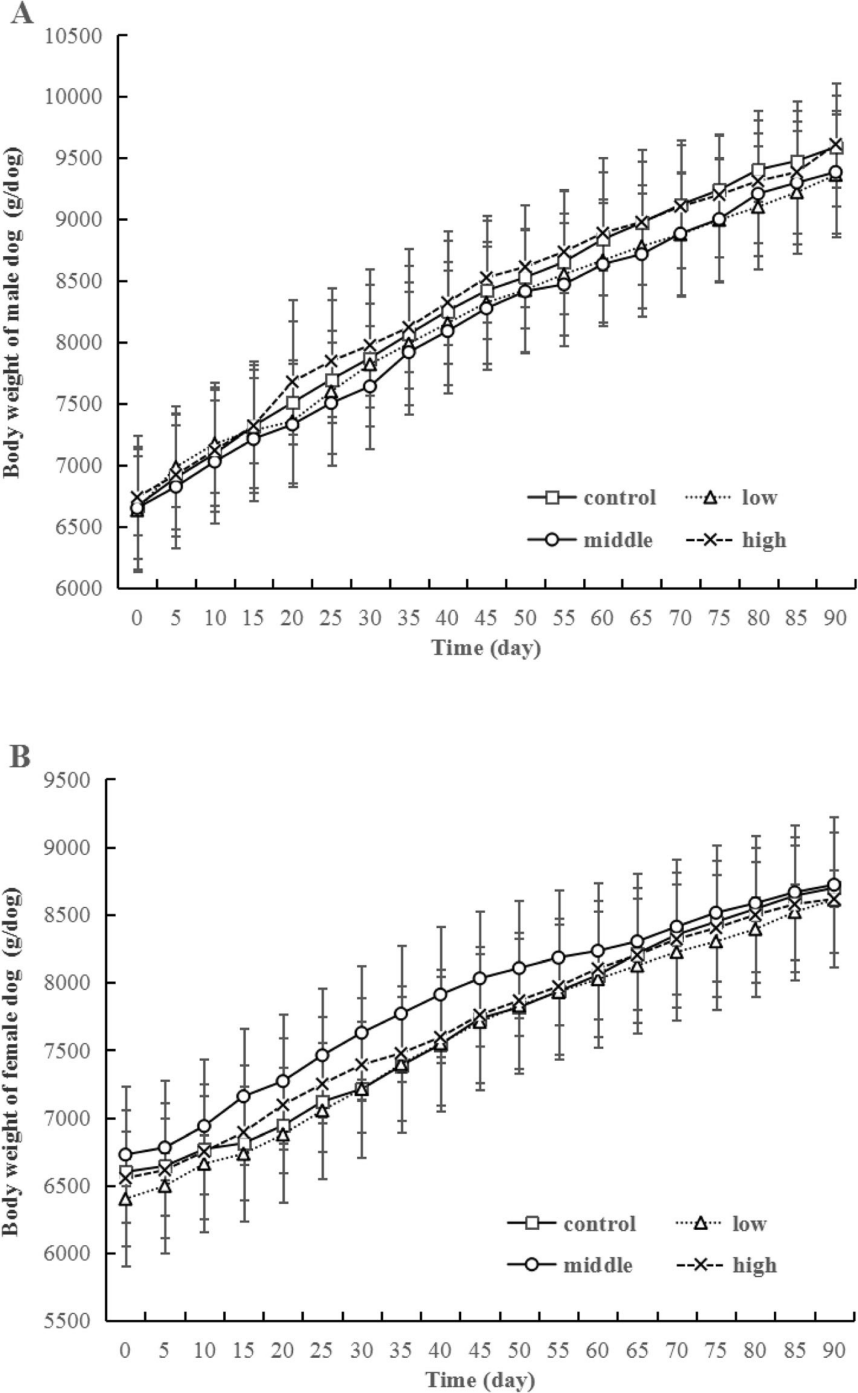
Body weights of beagle dogs at each time points. The body weights of males and females showed a steady growth tendency. Control, Low, Middle and High refer to 0, 12, 60 and 300 mg/kg ethanamizuril dose. A for male dogs, B for female dogs

**Fig. 3:**
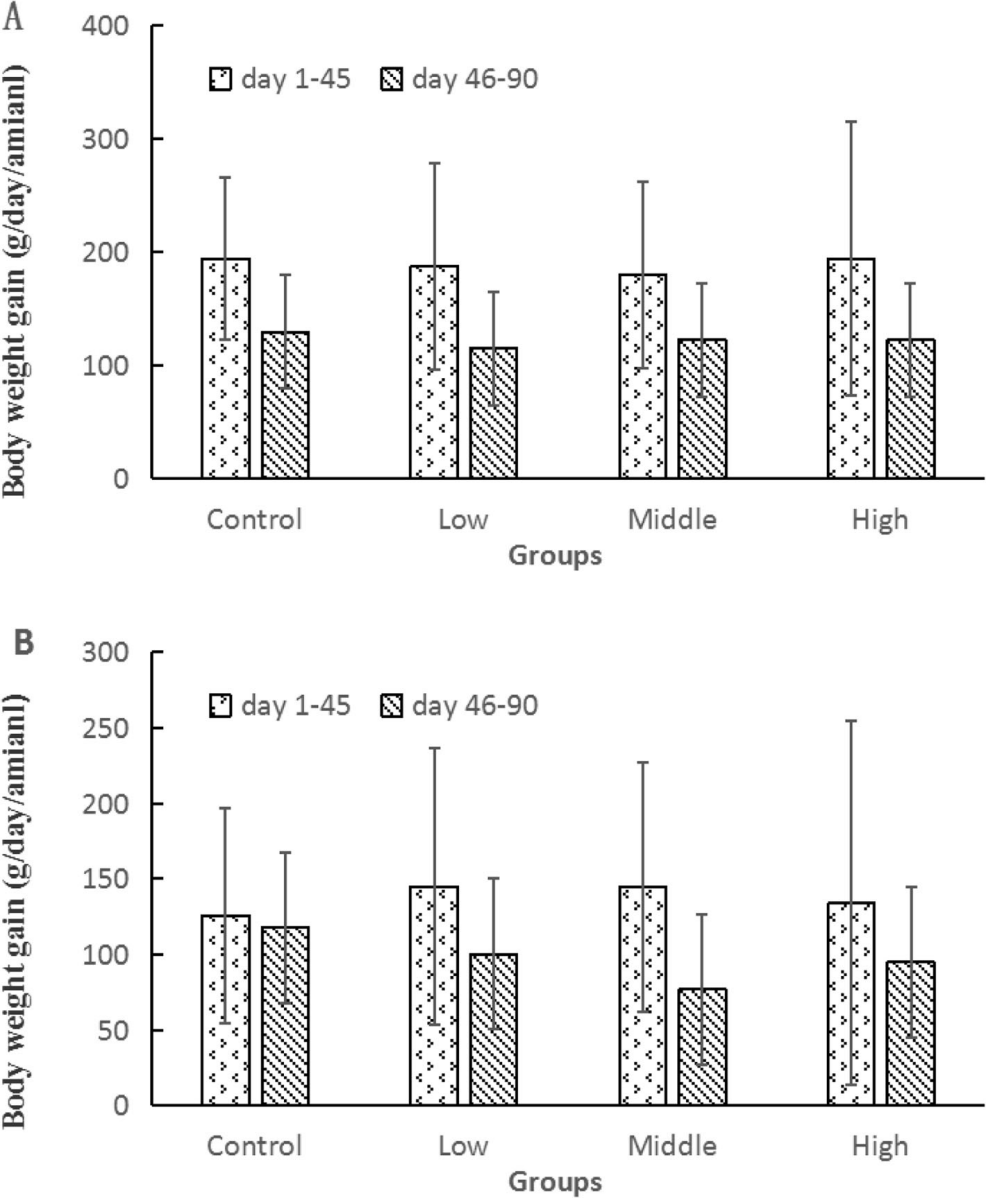
Body weight gains of beagle dogs at each time points. Low, Middle and High refer to 12, 60 and 300 mg/kg ethanamizuril dose. A for male dogs, B for female dogs

**Fig. 4:**
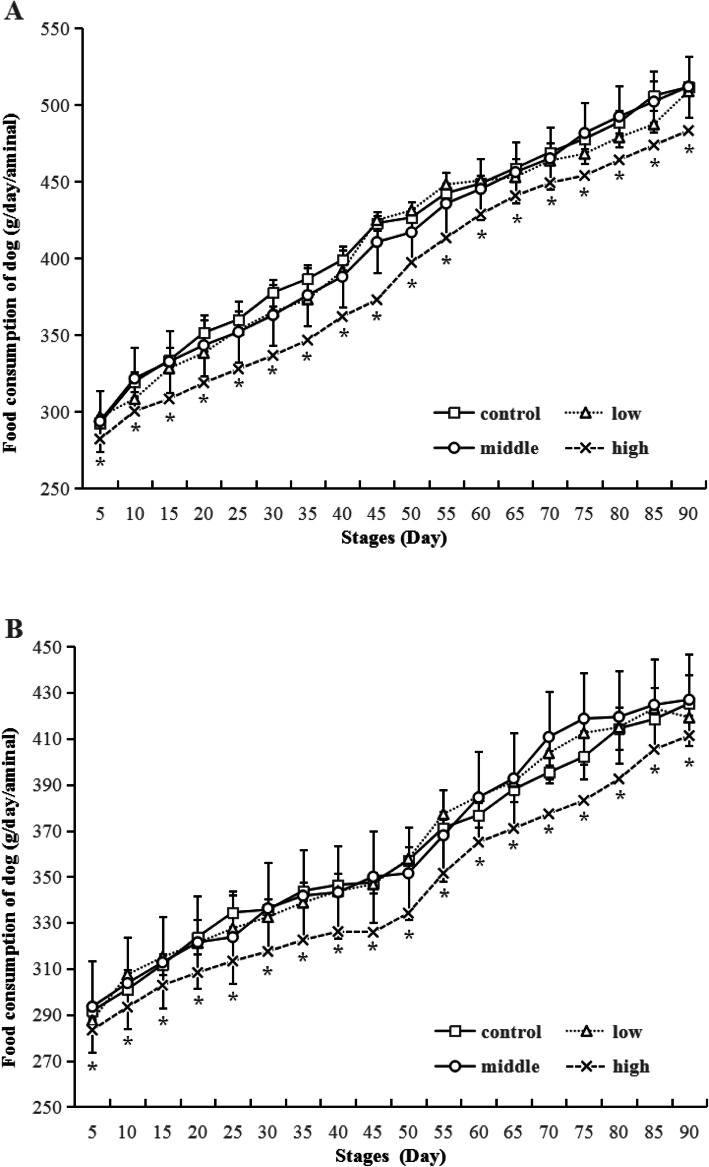
Food consumption of beagle dogs at each stages. Low, Middle and High refer to 12, 60 and 300 mg/kg ethanamizuril dose. A for male dogs, B for female dogs. * Significantly different from those of the control group at *p* < 0.05

The body weight changes of beagle dogs after ethanamizuril administration were shown in Fig. 2. The body weights of males and females showed a steady growth tendency and no significant statistical difference was noted at each time points. As shown in Table 1, the body weight of remaining dogs of control and high dose groups in convalescence showed no significant difference (the data were not analyze by statistics). No significant statistical difference was also observed for average body weight gains of all groups in each stage (Fig. 3). The weight gains of day 1–45 were higher than that of day 46–90, which showed a normal growth pattern of animals. A statistically significant reduction in food consumption was observed in the 300 mg/kg/day groups of both sexes compared to the control group in the whole treatment time (Fig. 4). Daily food consumed in the 300 mg/kg ethanamizuril group were 3–11.0% less than untreated control group. No other noted ethanamizuril related effects on food consumption or body weights were observed.

**Table 1:** The rate during the recovery period versus raw values in body weight of control and high dose groups in convalescence (Mean ± SD, %)

group	male		female	
	Control (***n*** = 2)	High (***n =*** 2)	Control (***n =*** 2)	High (***n =*** 2)
Day 95	3.46 ± 0.83	0.71 ± 3.54	0.27 ± 3.91	0.12 ± 5.22
Day 100	4.59 ± 1.42	1.91 ± 4.68	1.01 ± 3.55	1.40 ± 7.08
Day 105	5.93 ± 1.18	2.48 ± 4.58	2.30 ± 4.51	2.68 ± 7.55
Day 110	6.85 ± 0.31	3.13 ± 4.42	3.03 ± 4.59	2.91 ± 8.01
Day 115	7.59 ± 0.53	3.79 ± 5.32	3.57 ± 4.53	3.6 ± 8.71
Day 120	7.88 ± 0.34	4.93 ± 5.93	4.93 ± 5.86	4.21 ± 8.99

Note: The rate = 100%*(body weight in the recovery period - body weight in Day 90)/ body weight in Day 90. Control and high refer to 0 and 300 mg/kg ethanamizuril dose treated. No significant statistical difference was observed for the body weight

### Clinical pathology

The hematology, clinical biochemistry and urinalysis parameters of day 0, 45, 90 (scheduled necropsy), and 118 (end of convalescence) were analyzed in this study followed the test guideline 409 of OECD. The results showed that no significant ethanamizuril related changes in hematology and clinical biochemistry were noted in either males or females (Tables 2, 3). In addition, there were no ethanamizuril related effects noted on the evaluation of urinalysis parameters. It’s no statistically significant differences between the control and test ethanamizuril treated groups. Detection data of SG, pH and URO were presented in Table 4.

**Table 2:** Hematology of dogs on day 0, 45, 90 and 118

	Day 0				Day 45				day 90				day 118	
	High (***n =*** 6)	Middle (***n =*** 4)	Low(***n =*** 4)	Control(***n =*** 6)	High (***n =*** 6)	Middle(***n =*** 4)	Low(***n =*** 4)	Control(***n =*** 6)	High (***n =*** 6)	Middle(***n =*** 4)	Low(***n =*** 4)	Control (***n =*** 6)	High (***n =*** 2)	Control (***n =*** 2)
**Male**														
HGB (g/L)	159.70 ± 6.69	163.01 ± 6.39	163.72 ± 9.32	158.80 ± 6.46	160.35 ± 6.68	165.09 ± 5.45	167.66 ± 13.93	156.32 ± 9.10	158.97 ± 8.14	166.93 ± 0.75	167.66 ± 13.9	155.09 ± 10.17	166.52 ± 1.67	156.55 ± 5.20
RBC (10^12^/L)	7.66 ± 0.72	8.21 ± 0.30	8.03 ± 0.71	8.14 ± 0.62	7.52 ± 0.79	7.97 ± 0.54	8.02 ± 0.02	7.78 ± 0.55	7.38 ± 0.68	7.44 ± 0.10	8.02 ± 0.02	7.39 ± 0.21	7.35 ± 1.17	7.18 ± 0.18
WBC (10^9^/L)	13.21 ± 1.44	12.28 ± 1.44	12.29 ± 1.24	12.71 ± 1.51	12.70 ± 1.82	12.06 ± 1.39	12.14 ± 1.19	13.09 ± 1.52	11.99 ± 2.19	12.82 ± 1.52	12.14 ± 1.19	10.73 ± 0.45	13.90 ± 1.56	12.55 ± 2.40
PLT (10^9^/L)	670.20 ± 69.77	586.39 ± 7.75	687.71 ± 78.84	666.04 ± 91.36	649.40 ± 71.50	594.49 ± 98.53	710.70 ± 14.44	618.52 ± 69.15	668.92 ± 87.28	603.48 ± 24.83	710.70 ± 14.11	602.70 ± 79.93	392.35 ± 26.74	406.25 ± 136.81
HCT (%)	47.88 ± 2.61	48.05 ± 3.58	46.59 ± 1.24	45.65 ± 1.84	47.72 ± 4.10	39.96 ± 5.63	47.35 ± 0.55	46.45 ± 1.88	46.83 ± 1.99	48.66 ± 2.88	47.35 ± 0.55	45.00 ± 2.45	44.01 ± 1.0	43.00 ± 3.0
EOS (10^9^/L)	1.11 ± 0.08	1.13 ± 0.03	1.11 ± 0.02	1.09 ± 0.04	1.13 ± 0.07	1.14 ± 0.08	1.26 ± 0.09	1.19 ± 0.15	1.16 ± 0.11	1.13 ± 0.05	1.26 ± 0.09	1.19 ± 0.25	1.69 ± 0.06	1.92 ± 0.09
BAS (10^9^/L)	0.11 ± 0.04	0.13 ± 0.04	0.15 ± 0.04	0.14 ± 0.04	0.09 ± 0.07	0.15 ± 0.07	0.12 ± 0.09	0.11 ± 0.04	0.16 ± 0.04	0.20 ± 0.06	0.12 ± 0.09	0.11 ± 0.08	0.09 ± 0.01	0.08 ± 0.01
NEU (10^9^/L)	1.65 ± 0.88	2.19 ± 0.31	2.42 ± 0.52	2.25 ± 0.70	1.41 ± 0.83	1.83 ± 0.83	2.46 ± 0.21	1.78 ± 0.48	1.65 ± 0.74	1.58 ± 0.03	2.46 ± 0.21	1.77 ± 0.24	1.30 ± 0.39	1.67 ± 0.22
MO (10^9^/L)	3.28 ± 0.12	3.26 ± 0.02	3.22 ± 0.13	3.19 ± 0.13	3.30 ± 0.11	3.28 ± 0.03	3.35 ± 0.05	3.25 ± 0.15	3.32 ± 0.09	3.32 ± 0.03	3.35 ± 0.05	3.32 ± 0.15	3.94 ± 0.14	3.92 ± 0.09
LYMPH (10^9^/L)	15.02 ± 0.44	14.53 ± 0.63	14.87 ± 0.44	14.69 ± 0.82	15.23 ± 0.46	14.70 ± 0.58	14.73 ± 0.83	14.77 ± 0.87	15.29 ± 0.47	14.60 ± 0.18	14.73 ± 0.83	14.43 ± 1.23	15.05 ± 0.81	14.82 ± 0.77
**Female**														
HGB (g/L)	156.43 ± 6.99	165.17 ± 8.09	159.22 ± 14.25	160.26 ± 8.61	159.72 ± 6.46	163.54 ± 5.86	167.66 ± 14.01	160.17 ± 8.64	158.04 ± 6.94	159.74 ± 9.13	167.66 ± 14.01	157.24 ± 11.55	165.02 ± 11.04	165.02 ± 11.04
RBC (10^12^/L)	7.61 ± 0.74	7.90 ± 0.78	8.02 ± 0.72	7.90 ± 0.60	7.72 ± 0.76	8.01 ± 0.50	7.54 ± 0.68	7.91 ± 0.59	7.60 ± 0.42	7.67 ± 0.87	7.54 ± 0.68	7.84 ± 0.33	7.51 ± 0.73	7.20 ± 0.16
WBC (10^9^/L)	13.66 ± 1.04	11.91 ± 1.57	12.64 ± 1.31	13.00 ± 1.65	13.39 ± 1.69	12.28 ± 1.81	12.89 ± 1.16	12.97 ± 1.60	12.08 ± 1.90	11.43 ± 0.72	12.89 ± 1.16	12.73 ± 2.96	12.67 ± 2.87	12.35 ± 2.45
PLT (10^9^/L)	701.46 ± 72.18	669.34 ± 90.59	667.61 ± 62.86	667.79 ± 83.58	682.98 ± 87.40	618.30 ± 59.51	659.86 ± 71.32	639.10 ± 71.15	653.87 ± 89.75	688.37 ± 91.20	659.86 ± 71.32	604.53 ± 61.83	427.35 ± 148.73	395.86 ± 133.91
HCT (%)	47.34 ± 2.69	46.51 ± 2.54	48.63 ± 2.74	46.61 ± 2.45	46.93 ± 2.28	39.66 ± 5.44	45.11 ± 4.37	46.45 ± 1.95	46.13 ± 3.83	45.93 ± 4.04	45.11 ± 4.37	45.96 ± 1.57	42.01 ± 5.0	41.00 ± 2.0
EOS (10^9^/L)	1.13 ± 0.09	1.16 ± 0.14	1.13 ± 0.01	1.10 ± 0.07	1.14 ± 0.10	1.11 ± 0.05	1.20 ± 0.08	1.07 ± 0.07	1.14 ± 0.12	1.12 ± 0.03	1.20 ± 0.08	1.12 ± 0.03	1.39 ± 0.06	1.04 ± 0.14
BAS (10^9^/L)	0.11 ± 0.06	0.16 ± 0.14	0.14 ± 0.03	0.13 ± 0.03	0.09 ± 0.06	0.15 ± 0.05	0.11 ± 0.02	0.12 ± 0.14	0.10 ± 0.06	0.22 ± 0.11	0.11 ± 0.02	0.19 ± 0.11	0.09 ± 0.10	0.05 ± 0.03
NEU (10^9^/L)	1.71 ± 0.97	1.69 ± 0.34	2.37 ± 1.15	2.13 ± 0.63	1.66 ± 0.7	1.72 ± 0.12	2.43 ± 0.18	2.07 ± 0.66	1.74 ± 0.79	1.81 ± 0.46	2.43 ± 0.18	2.18 ± 0.64	2.00 ± 1.22	1.59 ± 0.21
MO (10^9^/L)	3.33 ± 0.9	3.29 ± 0.07	3.25 ± 0.08	3.26 ± 0.15	3.28 ± 0.08	3.24 ± 0.08	3.23 ± 0.13	3.26 ± 0.10	3.26 ± 0.06	3.25 ± 0.15	3.23 ± 0.13	3.24 ± 0.14	4.05 ± 0.15	4.04 ± 0.14
LYMPH (10^9^/L)	15.03 ± 0.43	14.73 ± 0.66	14.44 ± 0.74	14.76 ± 0.83	15.03 ± 0.50	14.46 ± 0.28	15.04 ± 0.96	14.95 ± 0.87	15.32 ± 0.49	14.96 ± 0.79	15.04 ± 0.96	14.52 ± 1.18	15.06 ± 0.81	14.86 ± 0.80

Note: Control, Low, Middle and High refer to 0, 12, 60 and 300 mg/kg ethanamizuril dose. There were no statistically significant differences when the control and test article-treated groups were compared

**Table 3:** Clinical chemistry of dogs on day 0, 45, 90 and 118

	Day 0				Day 45				day 90				day 118	
	High (***n*** = 6)	Middle (***n*** = 4)	Low(***n*** = 4)	Control (***n*** = 6)	High (***n*** = 6)	Middle (***n*** = 4)	Low (***n*** = 4)	Control (***n*** = 6)	High (***n*** = 6)	Middle (***n*** = 4)	Low (***n*** = 4)	Control (***n*** = 6)	High (***n*** = 2)	Control (***n*** = 2)
**Male**														
Alb (mmol/L)	35.36 ± 2.16	35.42 ± 1.68	36.21 ± 3.89	36.07 ± 2.18	34.87 ± 1.75	36.49 ± 3.70	36.17 ± 3.82	36.67 ± 2.27	35.25 ± 1.65	35.75 ± 2.40	36.03 ± 1.98	36.20 ± 1.89	34.99 ± 1.24	35.71 ± 2.43
ALT (U/L)	53.72 ± 6.47	56.87 ± 6.92	53.81 ± 5.10	57.92 ± 6.33	55.25 ± 6.62	54.40 ± 1.76	52.36 ± 2.56	57.66 ± 5.50	54.74 ± 5.93	51.90 ± 3.83	54.88 ± 6.36	55.44 ± 5.21	54.03 ± 7.75	58.77 ± 8.10
AST (U/L)	219.07 ± 26.45	206.39 ± 36.90	253.82 ± 31.42	248.25 ± 37.59	226.79 ± 27.84	218.28 ± 12.53	255.55 ± 28.06	233.24 ± 32.73	220.40 ± 30.74	235.21 ± 49.83	235.09 ± 30.69	225.90 ± 37.02	275.27 ± 34.93	226.48 ± 58.58
BUN (mmol/L)	5.06 ± 1.21	5.25 ± 1.39	4.85 ± 0.51	4.22 ± 0.88	5.25 ± 1.19	5.16 ± 1.09	5.33 ± 0.67	4.56 ± 0.74	5.63 ± 0.61	4.52 ± 0.94	5.07 ± 0.53	5.08 ± 0.71	4.22 ± 1.85	4.87 ± 1.13
TCH (mmol/L)	1.41 ± 0.25	1.58 ± 0.33	1.54 ± 0.28	1.59 ± 0.23	1.37 ± 0.29	1.35 ± 0.28	1.44 ± 0.26	1.61 ± 0.25	1.31 ± 0.35	1.64 ± 0.17	1.49 ± 0.23	1.64 ± 0.11	1.44 ± 0.60	1.65 ± 0.20
Cr (umoL/L)	42.93 ± 3.31	40.01 ± 9.07	47.35 ± 2.92	45.48 ± 2.92	45.00 ± 2.29	39.76 ± 4.47	47.33 ± 2.92	45.60 ± 2.50	44.70 ± 2.61	42.92 ± 8.47	45.02 ± 6.74	46.32 ± 4.36	42.67 ± 0.70	45.72 ± 4.54
Glu (mmol/L)	4.50 ± 0.91	4.27 ± 1.07	5.61 ± 1.01	5.22 ± 0.86	4.29 ± 1.20	4.46 ± 1.26	5.56 ± 1.03	5.12 ± 0.59	4.17 ± 1.00	4.78 ± 0.61	4.89 ± 0.50	4.91 ± 0.53	5.55 ± 0.21	5.05 ± 0.46
TP (G/L)	75.05 ± 2.72	76.01 ± 1.66	75.02 ± 4.60	72.64 ± 3.10	75.07 ± 1.28	75.70 ± 3.35	74.99 ± 4.52	73.35 ± 2.66	74.70 ± 0.74	74.99 ± 2.84	76.01 ± 3.14	75.24 ± 3.29	75.09 ± 1.49	72.49 ± 0.60
TG (mmol/L)	1.42 ± 0.06	1.40 ± 0.04	1.43 ± 0.03	1.39 ± 0.06	1.43 ± 0.06	1.42 ± 0.10	1.42 ± 0.05	1.38 ± 0.05	1.46 ± 0.10	1.43 ± 0.08	1.43 ± 0.09	1.39 ± 0.06	1.46 ± 0.02	1.40 ± 0.04
**Female**														
Alb (mmol/L)	35.30 ± 2.17	35.39 ± 2.16	36.36 ± 1.83	35.41 ± 2.12	35.36 ± 2.11	36.92 ± 1.52	36.10 ± 2.06	34.85 ± 2.06	34.62 ± 2.38	36.85 ± 2.33	35.59 ± 1.07	36.07 ± 1.23	36.40 ± 4.95	35.71 ± 2.30
ALT (U/L)	52.75 ± 6.31	52.31 ± 8.50	59.00 ± 10.26	57.66 ± 7.79	56.12 ± 7.66	51.37 ± 3.48	59.02 ± 10.33	60.49 ± 5.77	53.08 ± 7.40	56.82 ± 8.20	57.08 ± 6.03	56.16 ± 6.25	42.91 ± 0.14	58.76 ± 8.17
AST (U/L)	224.24 ± 30.90	225.92 ± 38.31	286.14 ± 39.24	239.81 ± 38.82	212.03 ± 14.14	247.89 ± 35.59	239.62 ± 5.35	240.87 ± 32.28	214.61 ± 19.90	226.69 ± 36.42	230.00 ± 33.50	242.82 ± 30.21	247.70 ± 46.71	226.45 ± 48.51
BUN (mmol/L)	5.35 ± 1.14	5.06 ± 1.16	4.71 ± 0.16	4.65 ± 0.66	5.11 ± 1.18	4.85 ± 0.81	3.68 ± 1.51	4.18 ± 0.87	5.27 ± 0.90	4.81 ± 0.49	4.35 ± 0.60	4.90 ± 0.58	5.67 ± 0.58	4.85 ± 1.09
TCH (mmol/L)	1.56 ± 0.50	1.49 ± 0.36	1.52 ± 0.46	1.64 ± 0.28	1.42 ± 0.43	1.43 ± 0.24	1.48 ± 0.54	1.60 ± 0.28	1.35 ± 0.16	1.44 ± 0.27	1.58 ± 0.15	1.66 ± 0.23	1.40 ± 0.24	1.65 ± 0.12
Cr (umoL/L)	44.75 ± 2.73	46.15 ± 3.65	44.45 ± 3.59	44.17 ± 4.06	43.90 ± 2.06	46.51 ± 4.38	44.38 ± 3.59	45.14 ± 3.15	42.57 ± 3.34	43.85 ± 5.03	46.65 ± 1.93	45.38 ± 2.46	40.09 ± 2.18	45.76 ± 4.44
Glu (mmol/L)	4.40 ± 1.16	4.29 ± 1.20	5.97 ± 0.42	5.37 ± 0.84	4.56 ± 0.63	5.00 ± 1.58	5.94 ± 0.51	5.16 ± 0.86	4.31 ± 1.12	4.93 ± 0.89	5.28 ± 0.70	4.74 ± 0.46	4.70 ± 2.13	5.03 ± 0.48
TP (G/L)	76.22 ± 3.76	75.17 ± 1.60	73.14 ± 3.68	74.29 ± 3.56	75.77 ± 2.03	76.31 ± 2.58	73.07 ± 3.61	73.35 ± 2.81	75.46 ± 1.26	74.11 ± 3.32	72.87 ± 2.04	71.97 ± 2.37	74.89 ± 0.98	72.46 ± 0.51
TG (mmol/L)	1.44 ± 0.09	1.43 ± 0.06	1.48 ± 0.01	1.40 ± 0.07	1.40 ± 0.14	1.36 ± 0.01	1.46 ± 0.05	1.42 ± 0.07	1.40 ± 0.03	1.36 ± 0.13	1.39 ± 0.05	1.34 ± 0.04	1.40 ± 0.04	1.41 ± 0.08

Note: Control, Low, Middle and High refer to 0, 12, 60 and 300 mg/kg ethanamizuril dose. There were no statistically significant differences when the control and test article-treated groups were compared

**Table 4:** Urinalysis of dogs on day 0, 45, 90 and 118

	male			female		
	SG	pH	URO	SG	pH	URO
**Day 0**						
High (*n =* 6)	1.08 ± 0.03	7.60 ± 0.32	3.11 ± 0.13	1.04 ± 0.04	7.44 ± 0.51	3.15 ± 0.08
Middle (*n =* 4)	1.09 ± 0.08	7.74 ± 0.56	3.07 ± 0.08	1.09 ± 0.15	7.39 ± 0.31	3.10 ± 0.09
Low(*n =* 4)	1.11 ± 0.04	7.49 ± 0.45	3.09 ± 0.06	1.09 ± 0.04	7.33 ± 0. 42	3.17 ± 0. 06
Control(*n =* 6)	1.05 ± 0.02	7.29 ± 0.27	3.09 ± 0.07	1.13 ± 0.08	7.49 ± 0.27	3.17 ± 0.06
**Day 45**						
High (*n =* 6)	1.07 ± 0.05	7.32 ± 0.33	3.11 ± 0.13	1.11 ± 0.11	7.41 ± 0.53	3.15 ± 0.08
Middle (*n =* 4)	1.09 ± 0.05	7.30 ± 0.14	3.10 ± 0.07	1.03 ± 0.10	7.42 ± 0.34	3.12 ± 0.03
Low(*n =* 4)	1.00 ± 0.03	7.47 ± 0.38	3.13 ± 0.09	1.06 ± 0.10	7.34 ± 0. 17	3.19 ± 0. 03
Control(*n =* 6)	1.05 ± 0.06	7.31 ± 0.25	3.08 ± 0.10	1.15 ± 0.08	7.37 ± 0.28	3.17 ± 0.10
**Day 90**						
High (*n =* 6)	1.09 ± 0.05	7.46 ± 0.42	3.13 ± 0.12	1.06 ± 0.03	7.26 ± 0.53	3.13 ± 0.07
Middle (*n =* 4)	1.13 ± 0.10	7.19 ± 0.12	3.05 ± 0.13	1.08 ± 0.06	7.31 ± 0.33	3.11 ± 0.09
Low(*n =* 4)	1.04 ± 0.07	7.55 ± 0.35	3.15 ± 0.01	1.10 ± 0.07	7.58 ± 0.36	3.16 ± 0. 04
Control(*n =* 6)	1.08 ± 0.07	7.39 ± 0.12	3.12 ± 0.10	1.18 ± 0.04	7.35 ± 0.25	3.13 ± 0.02
**Day 118**						
High (*n =* 2)	1.10 ± 0	7.48 ± 0.12	3.08 ± 0.06	1.03 ± 0.06	7.29 ± 0.80	3.17 ± 0.04
Control(*n =* 2)	1.08 ± 0.07	7.35 ± 0.30	3.08 ± 0.06	1.09 ± 0.01	7.74 ± 0.01	3.15 ± 0.02

Note: Control, Low, Middle and High refer to 0, 12, 60 and 300 mg/kg ethanamizuril dose. There were no statistically significant differences when the control and test article-treated groups were compared

### Macroscopic examination and organ weights

Dogs were euthanized on day 90 and 118 separately. The euthanasia method was abdominal aorta bleeding to death after being anesthetized with 50 mg/kg pentobarbital sodium. The organs or tissues of the animals were necropsied with macroscopic inspection. No ethanamizuril related systemic changes were observed in macroscopic inspection when the animals treated with 12, 60 and 300 mg/kg/day ethanamizuril. In addition, compared to the control group, there were no significant differences on the organ weights in either males or females when animals treated with 12, 60 and 300 mg/kg/day ethanamizuril. Summary data for organ relative weight were presented in Table 5.

**Table 5:** Relative organ weights (g/100 g final bw) in dogs fed ethanamizuril on day 90 and 118 in subchronic toxicity study (Mean ± SD)

	Day 90				Day 118	
	High (***n =*** 4)	Middle (***n =*** 4)	Low(***n =*** 4)	Control (***n =*** 4)	High (***n =*** 2)	Control (***n =*** 2)
**male**						
liver	48.1 ± 2.58	49.19 ± 1.77	50.26 ± 0.79	48.66 ± 2.15	50.02 ± 0.88	50.43 ± 0.31
kidney	4.63 ± 0.15	4.9 ± 0.27	5.01 ± 0.65	4.81 ± 0.12	4.55 ± 0.12	4.56 ± 0.20
spleen	4.19 ± 0.18	4.35 ± 0.22	4.31 ± 0.22	4.18 ± 0.28	4.17 ± 0.04	4.23 ± 0.10
stomach and intestine	84.93 ± 4.51	85.17 ± 3.93	84.84 ± 8.21	85.33 ± 4.67	79.61 ± 0.35	79.37 ± 2.74
lung	8.92 ± 0.22	9.12 ± 0.16	9.27 ± 0.45	9.08 ± 0.35	9.04 ± 0.13	9.22 ± 0.43
heart	8.86 ± 0.47	9.06 ± 0.39	10.10 ± 0.15	8.90 ± 0.18	8.85 ± 0.24	8.83 ± 0.11
brain	7.81 ± 0.23	8.16 ± 0.72	8.04 ± 0.25	7.97 ± 0.37	7.67 ± 0.12	7.60 ± 0.26
adrenal gland	0.20 ± 0.02	0.21 ± 0.02	0.22 ± 0.02	0.22 ± 0.01	0.16 ± 0.01	0.16 ± 0.01
testes	0.94 ± 0.09	0.93 ± 0.04	0.95 ± 0.04	0.94 ± 0.06	1.08 ± 0.06	1.01 ± 0.15
epididymides	0.36 ± 0.03	0.38 ± 0.03	0.31 ± 0.01	0.3 ± 0.03	0.30 ± 0.02	0.30 ± 0
**female**						
liver	47.81 ± 1.49	48.65 ± 2.26	48.8 ± 1.43	48.15 ± 2.12	46.13 ± 0.95	47.26 ± 2.66
kidney	4.88 ± 0.26	4.89 ± 0.19	5.15 ± 0.31	4.91 ± 0.20	4.88 ± 0.06	4.93 ± 0.39
spleen	3.83 ± 0.10	3.9 ± 0.16	3.89 ± 0.19	3.86 ± 0.12	3.66 ± 0.05	3.78 ± 0.46
stomach and intestine	89.78 ± 2.10	91.43 ± 3.18	88.14 ± 4.81	88.79 ± 4.65	86.06 ± 0.55	84.43 ± 2.90
lung	9.81 ± 0.26	10.15 ± 0.19	10.16 ± 0.38	9.80 ± 0.41	9.67 ± 0.20	9.54 ± 0.32
heart	9.47 ± 0.42	9.61 ± 0.28	9.14 ± 0.55	9.57 ± 0.55	9.34 ± 0.34	9.59 ± 0.57
brain	8.17 ± 0.28	8.62 ± 0.19	8.34 ± 0.14	8.3 ± 0.31	8.14 ± 0.92	8.33 ± 0.38
adrenal gland	0.19 ± 0.02	0.19 ± 0.01	0.17 ± 0.02	0.19 ± 0.01	0.18 ± 0	0.18 ± 0.01
uterus	0.15 ± 0.02	0.15 ± 0.01	0.15 ± 0.01	0.15 ± 0.01	0.13 ± 0.02	0.13 ± 0.01
ovaries	0.55 ± 0.03	0.57 ± 0.01	0.57 ± 0.02	0.56 ± 0.02	0.53 ± 0.04	0.54 ± 0

Note: Control, Low, Middle and High refer to 0, 12, 60 and 300 mg/kg ethanamizuril dose. No test article-related effects were noted on the organ weights of the male and female animals treated with ethanamizuril compared to the control group in either males or females

### Microscopic examination

**Fig. 5:**
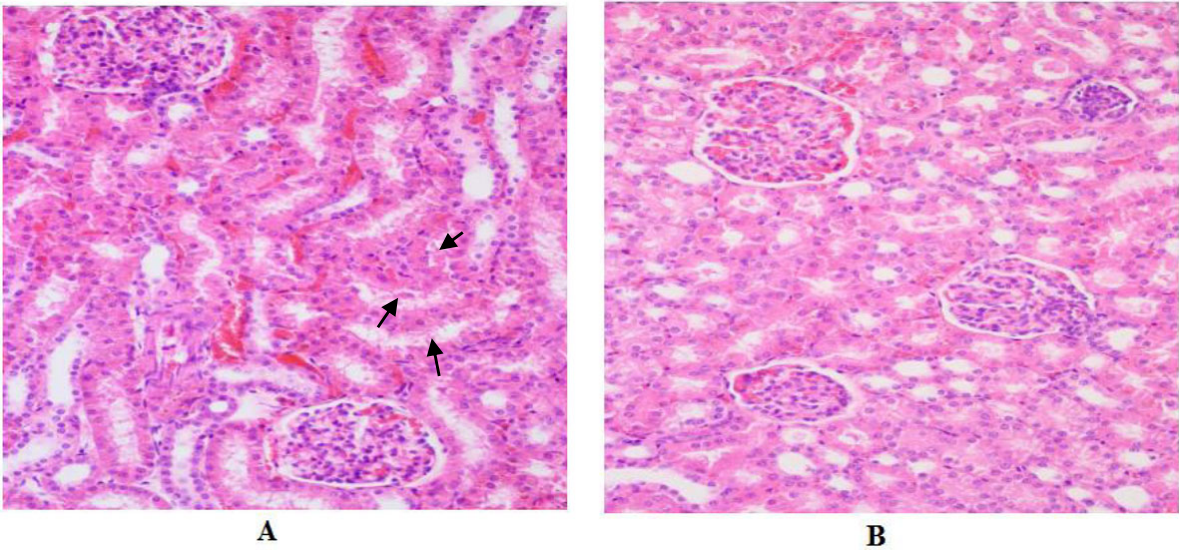
Slight congestion in renal tubulointerstitium was observed in the kidneys at 40X for the dogs treated with 300 mg/kg ethanamizuril in the 90 days chronic toxicity study with hematoxylin-eosin staining. (**a**) 300 mg/kg ethanamizuril treatment group, and → showed congestion in renal tubulointerstitium. (**b**) control group

Microscopic examination revealed increased incidence of slight congestion in renal tubulointerstitium for dogs of both sexes in the 300 mg/kg ethanamizuril group from day 90 after treatment (Fig. 5). However, the histologic changes of kidneys were recovery in dogs after drug withdrawal 4 weeks. There were no signs of ethanamizuril related histologic alterations appeared in other scheduled organs and tissues in the microscopic examination. The kidney was identified as the targets of a potential toxicity of ethanamizuril based on the results of this study.

## Discussion

Ethanamizuril, a novel coccidiostat, has potential high usage in the prevention and treatment of protozoal diseases in poultry industry. However, the toxicity in non-rodent species remains unclear. The current study is the first time to investigate a comprehensive toxicology of ethanamizuril in beagle dogs. Our results showed that daily administration of ethanamizuril at dose of 60 mg/kg are typical safe in Beagle dog.

In previous studies, we found that the NOAEL of ethanamizuril for rats was 20 mg/kg dietary dose level [10, 11]. Based on this result, we assumed that the dose of the dog would be greater than 60 mg/kg/day through the dose conversion with body surface area between different animals. In this study, dogs were administered ethanamizuril daily by diet for 90 consecutive days at dosage levels of 12, 60, and 300 mg/kg/day. During the experiment, no early animal death was observed, and no obvious behavior or external appearance changes related to ethanamizuril were observed. In addition, there were no ethanamizuril related impact on blood routine examination, serum chemistry, urine routine test, organ weights, and macroscopic evaluations. However, statistically significant reduced food consumption in the high-dose (300 mg/kg/day) group was observed. It’s speculate that a high concentration of ethanamizuril in the diet may cause the dogs feel discomfort and lead to slightly loss of appetite. Meanwhile, the histologic changes of kidneys noted in the high-dose (300 mg/kg/day) group in both sexes compared to controls, which may be related to treatment with ethanamizuril. Nevertheless the parameters related to renal function, such as BUN and creatinine, and urine analysis were not affected even in high-dose group. Furthermore, the tubules and the renal lesions caused by ethanamizuril were recovery after drug withdrawal.

With distinguished anticoccidial effectiveness, both diclazuril and toltrazuril are widely used to control coccidiosis of veterinary interest. Meanwhile, their toxicities have been concerned for a long time. In rat, there were slight effects on the haematological parameters and disturbances of the liver function in toxicity study of diclazuril and toltrazuril [11, 13, 14]. Beagle dogs administered 80 mg/kg bw/day diclazuril for 3 months displayed a fine granular, yellow to brown pigment in the cytoplasm of the hepatocytes [13]. In addition, the high dose of toltrazuril caused the weight of the testes and weight of the prostate decreased [13]. However, no obvious body weights, fetal body lengths, tail lengths, litter weights, number of viable fetuse, sexternal, skeletal or visceral malformations in fetuses were noted in any groups in two-generation reproduction and teratogenic test with ethanamizuril, and no adverse effects on the central nervous system, cardiovascular system, and respiratory system were showed in safety pharmacology test either [15, 17, 18]. Furthermore, the studies of 30 and 90-day subchronic toxicity with feeding ethanamizuril fed to SD rats revealed that the high dose of ethanamizuril, above 60 mg/kg dietary level, could cause minor damage to the liver, kidneys, and other organs, and induce alopecia [11, 12]. Moreover, the disorder on the hematologic and biochemical parameters was observed in rats when they were treated with high dose of ethanamizuril [11, 12]. Fortunately, the lesions induced by ethanamizuril in rats could be rehabilitated obviously after cessation of the drug [11, 12].

An enormous array of animal factors and environmental conditions affect the outcome of hematologic and clinical biochemistry analysis. In the present study, compared with the control group, there was no significant fluctuation in the indexes of hematological, serum chemistry, urinalysis, organ weights, macroscopic evaluations in beagle dogs. However, it should be to pay attention to the reduced food consumption and the histologic changes of kidneys that were observed in beagle dog when animal treated with the high-dose (300 mg/kg/day) ethanamizuril. Notably, in the rat subchronic toxicity study of ethanamizuril, renal tubular atrophy, tubule protein casts and interstitial cell hyperplasia in kidneys in 130 mg/kg dose groups were also reported previously [11, 12]. Our results further supported that the renal lesions were the major concerns among several different animal models, which suggests that the kidney is the main toxic target organ of ethanamizuril with a long-term use.

## Conclusions

In conclusion, the results of the 90-day toxicity investigation described here provide a comprehensive toxicity profile of ethanamizuril. The limited toxicity changes of ethanamizuril related were reduced appetite and histologic lesions in kidneys at the 300 mg/kg/day. Nonetheless, oral administration of ethanamizuril for 90 consecutive days were well tolerated in mature beagle dogs, when the dosage of ethanamizuril was less than 300 mg/kg/day. For this reason, it was considered that the no-observed-adverse-effect level (NOAEL) is 60 mg/kg/day.

## Methods

### Test materials

Ethanamizuril (CAS:1560840–75-6, C_18_H_16_N_4_O_4_, molecular weight 352.3 g/mol, purity 98.6%), N-(4-(4-(3,5-dioxo-4,5-dihydro-1,2,4-triazin-2-(3H)-yl)-2-methylphenoxy)phenyl)acetamide, was synthesized by Shanghai Veterinary Research Institute, Chinese Academy of Agricultural Science (Shanghai, P.R. China) and characterized by NMR, IR, LC-MS, and LC-UV methods (data were not shown).

### Animal receipt, acclimation and husbandry

Forty beagle dogs (20 males and 20 females), approximately 4–6 months of age, were obtained in good health from Xinglong Laboratory Animal Breeding Plant, Haidian District, Beijing (Batch No. SCXK (Jing) 2016–0003). Each animal was immunized as planned prior to the study. All animals were under 7 days acclimatization of the testing facility conditions prior to dose administration. At the initiation of ethanamizuril administration, body weights of dog ranged from 6.0–7.0 kg. Each group of animals was housed in a separate room maintained at 18–25 °C, with 30–70% relative humidity, natural ventilation, and a 12-h light-dark cycle. Each dog stayed and was fed in individual stainless steel cages measuring 100 cm in height, 100 cm in length, and 90 cm in width. Noise was controlled below 50 dB. The animals were fed 2 times daily with a medicated diet at 9:00 and 15:00. Distilled water was available ad libitum throughout the study. Regular opportunity for exercise and social interaction were allowed for all animals. The study was approved (20160105) by the Institutional Animal Care and Use Committee at Shanghai Veterinary Research Institute, Chinese Academy of Agricultural Sciences.

### Diet preparation

According to the requirements of different doses in the beagle dog 90-day oral toxicity study, the diets were mixed separately by group and ethanamizuril was evenly incorporated in basal diet respectively. The processing of diet was in the charge of Beijing Keao Xieli Feed company (Beijing Keao Xieli Feed Co., Ltd., Beijing, China), and the components of the diet include: water content ≤10%, crude protein ≥20%, crude fat ≥8%, Crude fiber ≤4%, Crude ash ≤9%, calcium 0.7–1.0%, Total phosphorus 0.5–0.8%. To ensure the homogeneity and effectiveness, the drugs were weighed carefully and were thoroughly mixed and prepared every 4 weeks. In addition, the stability and homogeneity of the diets were verified prior to the study by HPLC method [19].

### Assignment of animals to treatment groups

The animals were randomly divided into four groups by using of Excel software based on the body weight, and each group of dogs were fed basal diets mixed with 0, 12, 60 and 300 mg/kg ethanamizuril for a total period of 90 days, respectively. The low and middle dose groups each consisted of 4 males and 4 females, and the control and high dose groups each consisted of 6 males and 6 females. Animals were dosed for 90 days and four dogs/sex/group were sacrificed under anesthesia with sodium pentobarbital. The remaining dogs of control and high dose groups were administered control feed for a further 4 weeks (convalescence) after which they were killed in the same manner and subjected to examination.

### Parameters evaluated

#### Clinical observations

All animals in the study were observed at least twice daily for any changes in appearance of coat, activity and respiration, food and water intake, micturition and stool excretion. The presence or absence of findings in each animal was recorded regularly.

Prior to the start of ethanamizuril administration and at the end of the treatment period, ophthalmological examinations with fluorescein sodium method were performed respectively in control and high dose groups. If there were the changes of ophthalmology in the high dose group, all animals in the other dosing groups should be examined.

#### Body weights, food consumption

The individual body weight and food consumption weights of animals were recorded every 5 days throughout the study period. Food consumption was calculated as g/animal/day. In addition, the body weight on the day of randomization was also recorded.

#### Clinical pathology/laboratory examinations

To detect hematology and serum chemistry parameters, blood samples of treated animals were collected during study day 0, 45, 90 (scheduled necropsy), and 118 (end of convalescence). Prior to blood collection, animals were fasted overnight. Hematological test parameters were basophil (BAS), eosinophil (EOS), erythrocyte count (RBC), hematocrit (HCT), hemoglobin (HGB), leukocyte count (WBC), lymphocyte (LYMPH), monocytes (MO), neutrophils (NEU), platelet count (PLT). Albumin (Alb), alanine aminotransferase (ALT), blood glucose (Glu), blood urea nitrogen (BUN), creatinine (Cr), glutathione aminotransferase (AST), total cholesterol (TCH), total protein (TP), triglyceride (TG) were included in clinical chemistry test parameters.

During study day 0, 45, 90 (scheduled necropsy), and 118 (end of convalescence), urine samples were also collected from all animals by using cage pans. Bilirubin (T-BIL), glucose (GLU), ketones (KET), occult blood (BLO), protein (PRO) and white blood cells (WBC) were detected using qualitative indicators of analyte concentration. Urine pH, specific gravity (SG) and urobilinogen (URO) were measured quantitatively.

### Necropsy and pathology

After injected sodium pentobarbital with small saphenous vein, the dogs were euthanized by exsanguination via the abdominal aorta under anesthesia. Four dogs/sex/group were euthanized on day 90 and the remaining dogs of control and high dose groups were euthanized on day 118. The necropsy included, but was not limited to, examining the body external surface, all orifices, and all organs in coelom. All the organs from animals at the scheduled necropsy including the adrenals, brain, heart, kidney, liver, lung, spleen, stomach and intestine, testis and epididymides, ovaries and uterus were weighed. The organ relative weight (the percentage of organ weight to body weight) was calculated. Based on the SOPs of histopathology technical operation, the tissue sampling, paraffin embedding, sectioning, and hematoxylin-eosin staining were conducted for the above organs from each animal, and then evaluation via light microscopic for morphological alterations. Tissues from other groups were examined as necessary to determine NOAEL in target organs.

### Statistical methods

The treated groups were compared to their respective control groups. To determine intergroup differences, the data in the study were applied to a parametric one-way analysis of variance (ANOVA) [20]. When statistically significant (*p* < 0.05) intergroup variance was revealed by ANOVA, Dunnett’s test was applied to compare the groups.

## Supplementary Information

**Additional file 1.** The ARRIVE Guidelines Checklist.

## Data Availability

All data generated or analysed during this study are included in this published article [and its supplementary information files].

## Abbreviations

NOAEL: no-observed-adverse-effect level
AAALAC: Association for Assessment and Accreditation of Laboratory Animal Care
ANOVA: one-way analysis of variance
PRO: protein
GLU: glucose
T-BIL: bilirubin
URO: urobilinogen
BLO: occult blood
WBC: white blood cells
KET: ketones
HGB: hemoglobin
RBC: erythrocyte count
WBC: leukocyte count
PLT: platelet count
HCT: hematocrit
EOS: eosinophil
BAS: basophil
NEU: neutrophils
MO: monocytes
LYMPH: lymphocyte
Alb: albumin
ALT: alanine aminotransferase
AST: glutathione aminotransferase
BUN: blood urea nitrogen
TCH: total cholesterol
Cr: creatinine
Glu: blood glucose
TP: total protein
TG: triglyceride
